# Dual‐Phase C‐11 PiB PET Images for Detecting Tau Pathology in Cerebral Amyloid Angiopathy

**DOI:** 10.1002/acn3.70021

**Published:** 2025-03-03

**Authors:** Meng‐Ting Chiang, Chia‐Ju Liu, Bo‐Ching Lee, Ruoh‐Fang Yen, Hsin‐Hsi Tsai

**Affiliations:** ^1^ Department of Nuclear Medicine Taipei Medical University Hospital Taipei Taiwan; ^2^ Department of Nuclear Medicine National Taiwan University Hospital Taipei Taiwan; ^3^ Department of Medical Imaging National Taiwan University Hospital and National Taiwan University College of Medicine Taipei Taiwan; ^4^ Department of Neurology National Taiwan University Hospital Taipei Taiwan

**Keywords:** C‐11 Pittsburgh compound‐B positron emission tomography (C‐11 PiB PET), cerebral amyloid angiopathy (CAA), early‐phase amyloid PET, neurodegeneration, tau PET

## Abstract

**Background:**

Cerebral amyloid angiopathy (CAA) is a major cause of lobar intracerebral hemorrhage and cognitive dysfunction in the elderly, and frequently coexists with Alzheimer's disease and tau pathology. Dual‐phase ^11^C‐PiB PET detects amyloid deposition and cerebral perfusion changes and may have diagnostic value for identifying tau in CAA.

**Methods:**

We prospectively enrolled patients with probable CAA for dynamic PiB and AV1451 scans. We compared early‐phase (0–6 min after tracer injection) and late‐phase (40–70 min) PiB PET between the tau(+) and tau(−) groups (based on AV1451 PET) and investigated their diagnostic values for detecting tau.

**Results:**

CAA/tau(+) had lower early‐phase temporal PiB uptake than CAA/tau(−) (*p* = 0.014) and higher late‐phase uptake in the whole cortex and temporal and parietal lobes (all *p* < 0.05). Early‐phase temporal PiB SUVR correlated with tau burden (*r* = −0.34, *p* = 0.038). Using Youden's cut‐off, early‐phase and late‐phase PET had sensitivities of 55% and 80% and specificities of 85% and 65% for detecting tau, respectively. Combining early‐ and late‐phase scans provided a rule‐out sensitivity of 90% and rule‐in specificity of 100% for tau pathology in CAA.

**Conclusions:**

Dual‐phase ^11^C‐PiB PET represents a reliable approach for assessing tau and could potentially identify CAA patients for tau biomarker testing.

## 1 Introduction

Cerebral amyloid angiopathy (CAA) is the major vascular etiology involved in the development of spontaneous lobar intracerebral hemorrhage (ICH) and frequently co‐occurs with cognitive dysfunction, especially among elderly patients [1]. CAA involves the gradual deposition of β‐amyloid (Aβ) in the small‐to‐medium vessels at leptomeninges and in the cerebral cortex. A definitive diagnosis of CAA requires a full post‐mortem examination. In current clinical practice, most patients with CAA are diagnosed with “probable or possible CAA” based on the Boston criteria following blood‐sensitive magnetic resonance imaging (MRI) [2].

Molecular imaging by positron emission tomography (PET) provides in vivo information on the functional and pathological status of the brain. Radiotracers, such as amyloid PET agents like ^11^C‐Pittsburgh Compound‐B (PiB), can help to identify and assess the topographical distribution of amyloid deposition in CAA [3]. However, the diagnostic role of amyloid PET imaging remains uncertain, as CAA is commonly comorbid with Alzheimer's disease (AD) [4, 5] and the current tracers lack specificity to differentiate between vascular and parenchymal Aβ. The presence of tau pathology on PET is a potential marker of concomitant AD in CAA [6, 7]; however, unfortunately, tau PET tracers are not yet widely available at most PET centers.

Based on recent studies, early‐phase tracer uptake on amyloid PET has been proposed as an additional parameter of cerebral perfusion that closely correlates with parameters of brain metabolism [8, 9]. Therefore, a dual‐phase protocol for amyloid scans has been recommended to provide better diagnostic and prognostic yield than the conventional late‐scan only protocol [10, 11]. However, to date, only one study has evaluated the diagnostic value of early‐phase amyloid PET to differentiate between CAA and AD [12] and no studies have yet evaluated the relationship between dual‐phase PiB and concomitant tau pathology in patients with CAA. As markers of perfusion deficit may be suggestive of concomitant tau pathology and neurodegeneration in CAA, we anticipate that dual‐phase amyloid PET could be an effective screening tool to identify CAA patients who may require further testing for tau biomarkers, including tau PET or cerebrospinal fluid (CSF) analysis.

In the current prospective study, we aimed to compare early‐ and late‐phase PiB uptake in CAA between patients with and without tau pathology in the AD‐signature region (i.e., meta‐temporal regions of interest). In addition, we also investigated the diagnostic value of dual‐phase ^11^C‐PiB PET scans to predict tau pathology in patients with CAA.

## 2 Materials and Methods

### 2.1 Patients

**FIGURE 1:**
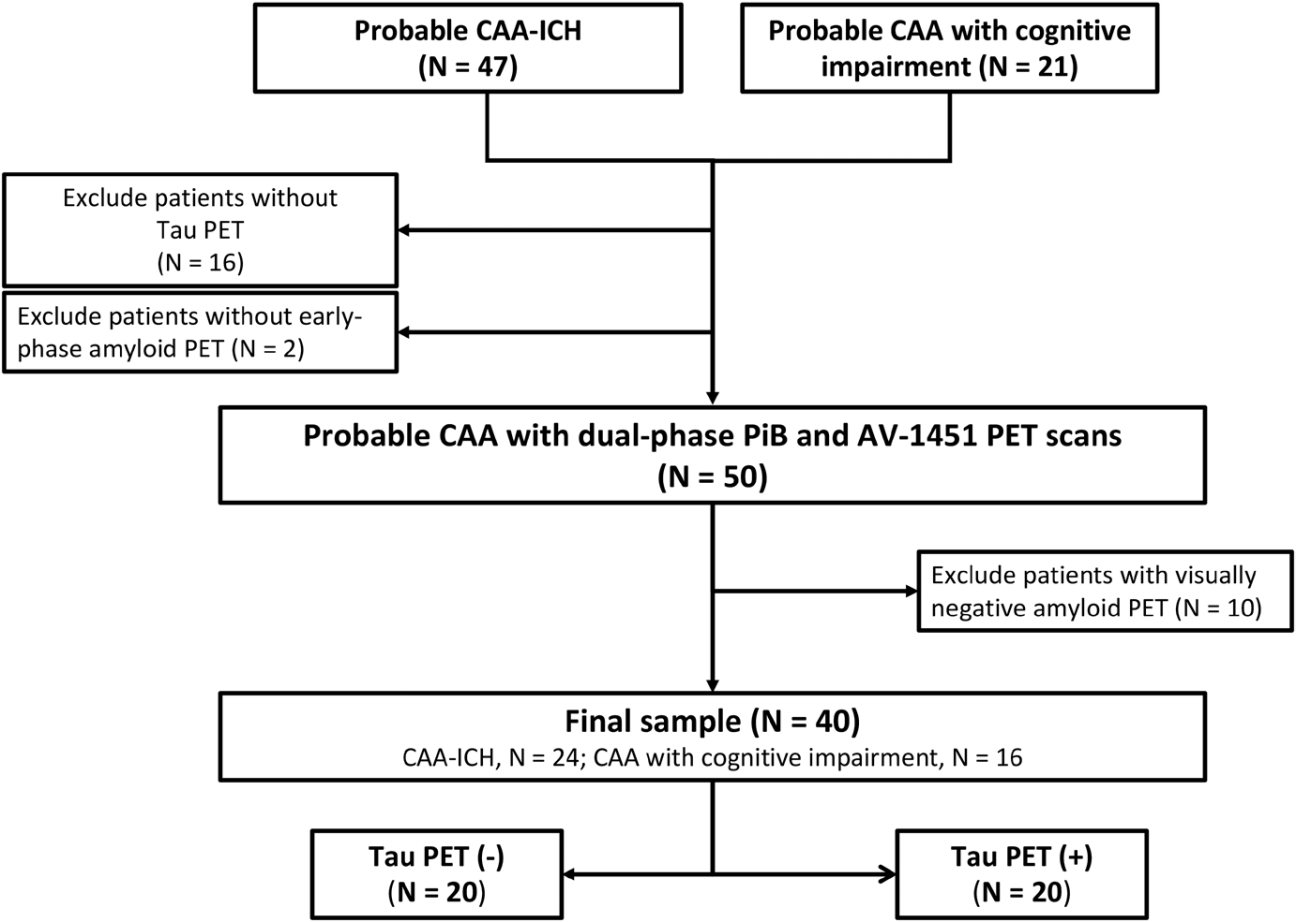
Patient enrollment flow chart.

We prospectively enrolled patients aged 55 years or older diagnosed with probable cerebral amyloid angiopathy (CAA) according to the Boston criteria version 1.5 (before version 2.0 was published) or version 2.0 who presented with spontaneous intracerebral hemorrhage (ICH) or attended the cognition clinic. A total of 47 Chinese patients with probable CAA from the ICH cohort and 21 patients with probable CAA from the cognition cohort were enrolled. Sixteen patients were excluded due to the unavailability of tau PET data, and two patients were excluded due to the unavailability of early‐phase amyloid PET data. Dual‐phase PiB and AV‐1451 tau PET images were successfully obtained for 50 patients with probable CAA. As an assessment of cerebral tau pathology is usually required for patients that present with amyloid pathology, ten cases with visually amyloid‐negative PET scans were excluded; thus, 40 patients (24 from the ICH cohort and 16 from the cognition cohort) were included in the final statistical analysis (Figure 1). This study complied with the Declaration of Helsinki and was approved by the Research Ethics Committee of our hospital (201903069RINB). Written informed consent was obtained from all participants or their family members.

### 2.2 MRI Acquisition and Analysis

Brain MRIs were performed at the Department of Medical Imaging using a 3‐Tesla scanner (Siemens Verio, TIM, or mMR; Siemens Medical Solutions, Malvern, PA, USA). The imaging protocols included T1‐weighted imaging, T2‐weighted imaging, fluid‐attenuated inversion recovery (FLAIR) imaging, susceptibility‐weighted imaging (SWI), diffusion‐weighted imaging (DWI), and apparent diffusion coefficient (ADC) maps. MRI markers related to cerebral small vessel disease (SVD) were evaluated according to the Standards for Reporting Vascular Changes on Neuroimaging V2.0 (STRIVE2) criteria [13].

The total CAA MRI small vessel disease score (range, 0 to 6) was used to quantify the burden of CAA based on evaluation of four key MRI markers that are characteristic of the disease: lobar cerebral microbleeds (CMBs), cortical superficial siderosis (cSS), centrum semiovale perivascular spaces (CSO‐PVSs), and white matter hyperintensities (WMHs) [14]. For lobar CMBs, a score of 1 point is assigned if two to four CMBs are present, and two points are assigned for the presence of five or more CMBs. The presence of cSS is scored as 1 point if focal and 2 points if disseminated. CSO‐PVSs are scored as 1 point if moderate‐to‐severe (grade 3–4, i.e., > 20) PVSs are present. WMHs are scored as 1 point if either early confluent deep WMHs (Fazekas score ≥ 2) or irregular periventricular WMHs extending into the deep white matter (Fazekas score of 3) are present [14].

The hippocampal volume and mean cortical thickness were assessed using T1‐weighted magnetization‐prepared rapid gradient‐echo (MPRAGE) imaging. Data were processed with FreeSurfer software v6.0.0, accessed via FreeSurfer's website.

### 2.3 PET/CT Acquisition and Analysis

^11^C‐PiB and ^18^F AV‐1451 PET tracers were manufactured and handled according to Good Manufacturing Practice at our PET Center. Static PET/CT scans (GE Discovery PET/CT 710 or Siemens Biograph mCT 20) were acquired in three‐dimensional mode. Scanning commenced immediately after injection of the tracer, with the dynamic PET emission scan lasting for 70 min (23 frames: four for 30 s, nine for 1 min, three for 3 min, four for 5 min, and three for 10 min). The first 6 min after bolus injection of 10 mCi of ^11^C‐PiB were reconstructed to obtain the early‐phase image [12] and the late‐phase image was derived from the 40th minutes to the last frame (up to the 70th minute). Static scanning for ^18^F‐ AV‐1451 PET images was acquired for 20 min, starting 80 min after injection of the radiotracer. All PET scanners were comparable. PET data were reconstructed using ordered subset expectation maximization (five iterations, 32 subsets, post‐filter 2.57) and corrected for attenuation. Each PiB or Tau PET image was realigned, resliced, and manually co‐registered to a standardized CT template using PMOD software. We used the AAL atlas to extract the regional SUVRs for the regions of interest (ROIs) using the cerebellum as a reference, as previously reported [7, 15].

The interpretation of positive amyloid PET scans was conducted by an experienced and board‐certified nuclear medicine specialist with over 5 years of reading expertise (author C.J. L) [16, 17]. A positive tau PET scan was operationally defined as a standardized uptake value ratio (SUVR) greater than 1.26 in the meta‐temporal region (entorhinal cortex, amygdala, parahippocampal gyrus, fusiform gyrus, and inferior and middle temporal gyri) [18], in accordance with the results for previous cohorts based on the mean and two standard deviations (SD) of tau uptake in the meta‐temporal ROI for amyloid‐negative and cognitively unimpaired participants [19].

Dual‐phase amyloid PET using ^11^C‐PiB and Tau PET using ^18^F‐ flortaucipir were performed within an interval of 2 to 16 weeks.

### 2.4 Statistical Analysis

Continuous variables are expressed as mean ± standard deviation and were compared using independent *t*‐tests or as median (interquartile range) and compared using Mann–Whitney tests. Categorical variables are presented as frequency and percentage and were analyzed using Fisher's exact test. As this was a hypothesis‐generating study, we did not adjust for multiple comparisons.

Single linear regression and multiple regression analyses, adjusted for age and late‐phase whole cortex PiB SUVR, were conducted to assess the correlations between variables using multivariable linear regression models adjusted for potential confounders. To assess the diagnostic value of the PET parameters, we performed receiver operating characteristic (ROC) analysis, and the optimal cut‐off values were determined using the Youden index. ROC analyses included the calculation of the area under the curve (AUC), sensitivity, specificity, positive predictive value (PPV), and negative predictive value (NPC). The ROC curves were compared using a pairwise comparison test developed by Delong et al. [20].

Statistical analysis was performed using MedCalc (version 22.021, Ostend, Belgium); *p*‐values < 0.05 were considered statistically significant.

## 3 Results

The demographic and clinical data for the study participants with CAA are presented in Table 1. The mean age was 75 ± 7.0 years, and 25 patients (62.5%) were female. The distribution of tau PET SUVR in the meta‐temporal ROI is shown in Figure S1. Twenty patients (50%) had tau‐positive PET scans, termed tau(+). No significant differences in age, sex, years of education, prevalence of hypertension, diabetes, hyperlipidemia, and *ApoE2* or *ApoE4* carrier status were observed between the tau(+) vs. tau(−) groups. However, as expected, the tau(+) group had significantly lower MMSE scores than the tau(−) group (17 ± 8.1 vs. 23 ± 7.8, *p* = 0.021; Table 1).

**TABLE 1:** Comparison of the demographic and radiological features of the CAA/tau(+) and CAA/tau(−) groups.

	Tau(+) (*n* = 20)	Tau(−) (*n* = 20)	*p*
Female, %	12 (60.0%)	13 (65.0%)	1.000
Age, years	75.0 (± 7.3)	74.8 (± 6.9)	0.935
Years of education	9.4 (± 4.0)	10.0 (± 5.0)	0.679
Hypertension, %	11 (55.0%)	14 (70.0%)	0.514
Diabetes, %	4 (20.0%)	3 (15.0%)	0.704
Hyperlipidemia, %	5 (25.0%)	4 (20.0%)	1.000
MMSE	17.3 (± 8.1)	23.4 (± 7.8)	0.021
Baseline CDR > 0.5, %	12 (60.0%)	6 (30.0%)	0.111
*ApoE2* carrier, %	8 (40.0%)	4 (20.0%)	0.301
*ApoE4* carrier, %	9 (45.0%)	7 (35.0%)	0.540
Cerebral microbleeds			
Lobar CMBs	5.5 (2.0–37.5)	4.0 (2.0–12.0)	0.634
Cerebellar CMBs	1.0 (0.0–8.0)	0.0 (0.0–1.0)	0.142
WMH volume, mL	4.4 (2.8–7.2)	4.3 (1.5–6.9)	0.735
Lacunes, %	7 (35.0%)	3 (15.0%)	0.273
MRI‐visible enlarged perivascular spaces			
Basal ganglia (> 20), %	6 (30.0%)	9 (45.0%)	0.514
Centrum semiovale (> 20), %	14 (70.0%)	11 (55.0%)	0.514
Cortical superficial siderosis, %	11 (55.0%)	7 (35.0%)	0.341
Total cSS score	1.0 (0.0–3.0)	0.0 (0.0–1.0)	0.024
CAA score	4 (2.5–4.5)	3 (2.0–4.0)	0.041
Hippocampal volume, mL^a^	3.3 (3.0–3.4)	3.6 (3.0–3.8)	0.036
Mean cortical thickness, mm^a^	2.19 (± 0.16)	2.30 (± 0.15)	0.040

*Note:* Values are mean (± standard deviation), median (IQR), or number (percentage).

Abbreviations: CAA, cerebral amyloid angiopathy, CMB, cerebral microbleeds; cSS, cortical superficial siderosis; IQR, interquartile range; MMSE, Mini‐Mental Status Exam; WMH, white matter hyperintensities.

^a^ Hippocampal volume and mean cortical thickness were not available for two patients.

On structural neuroimaging analysis, the tau(+) group exhibited higher total cSS scores (1 [0.0–3.0] vs. 0 [0.0–1.0], *p* = 0.024) and CAA scores (4 [2.5–4.5] vs. 3 [2.0–4.0], *p* = 0.041) than the tau(−) group. As expected, the tau(+) group had a lower hippocampal volume (3.3 [3.0–3.4] vs. 3.6 [3.0–3.8] mL, *p* = 0.036) and a lower mean cortical thickness (2.19 ± 0.16 vs. 2.30 ± 0.15 mm, *p* = 0.004; Table 1) than the tau(−) group.

### 3.1 Comparison of Early‐ and Late‐Phase Amyloid Uptake Between Tau(+) And Tau(−) CAA

**FIGURE 2:**
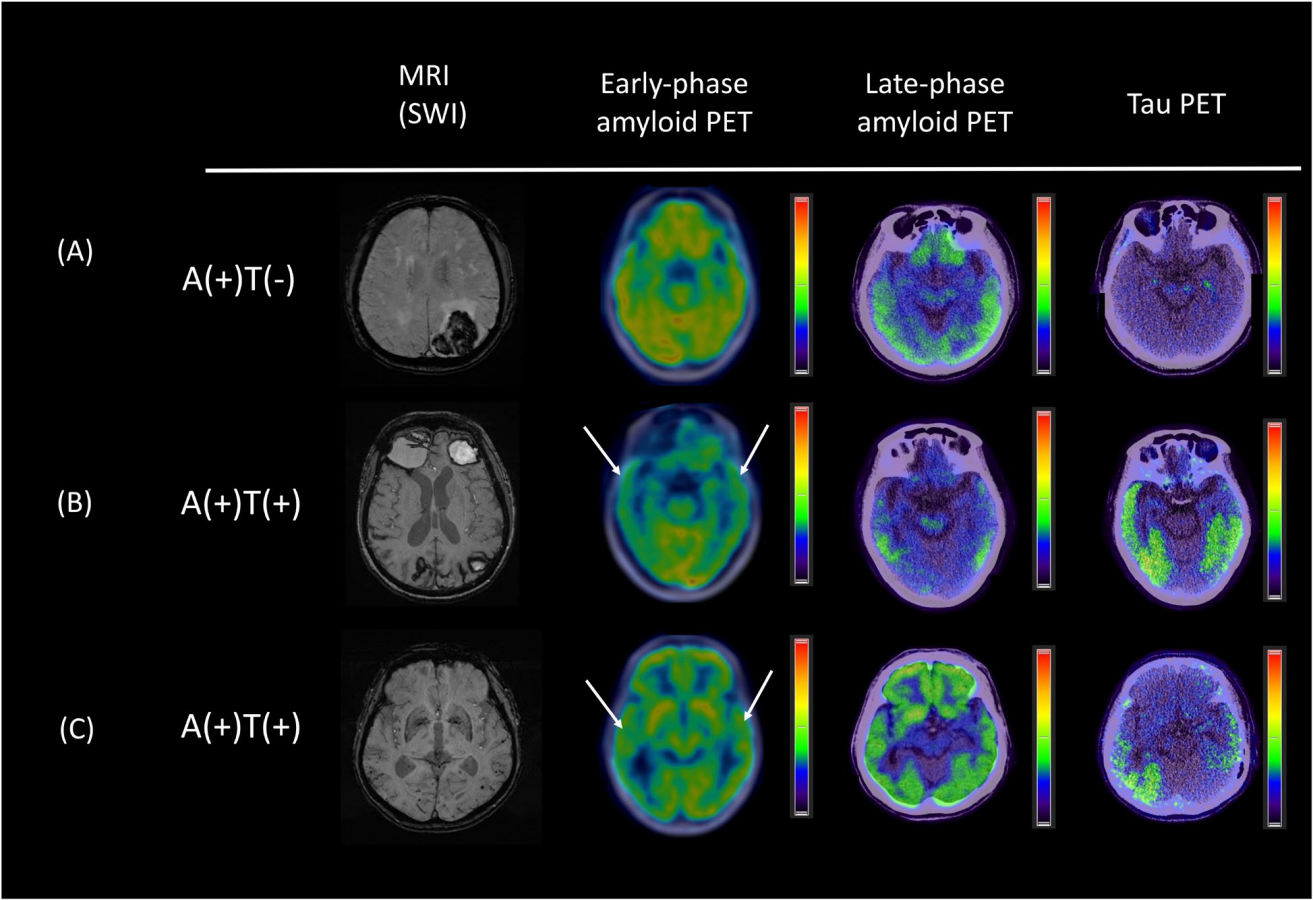
Representative neuroimaging of patients with CAA. (A) A tau(−) patient with CAA‐ICH who exhibited relatively intact perfusion status in the early‐phase amyloid scan. (B) A tau(+) patient with CAA‐ICH who exhibited reduced perfusion in the early‐phase scan; this reduction was most pronounced in the bilateral temporal lobes (arrows). (C) A CAA patient with cognitive impairment who had multiple lobar CMBs in the posterior brain. Positive tau pathology was found on tau PET. Mildly reduced perfusion in the bilateral temporal region was noted in the early‐phase amyloid scan (arrows). Note that all patients had visually positive late‐phase amyloid scans.

**FIGURE 3:**
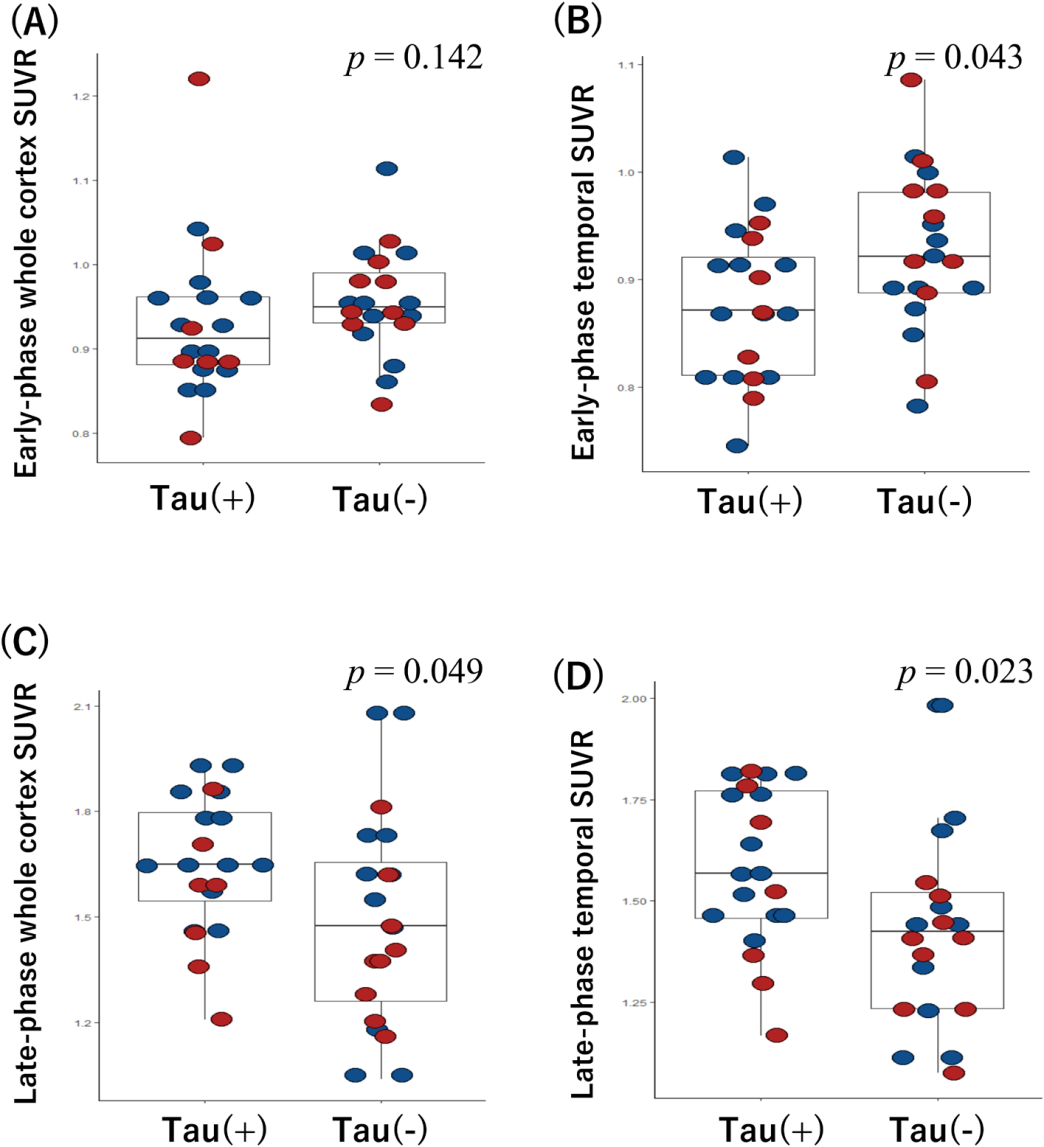
Comparison of early‐ and late‐phase PiB PET uptake between the CAA/tau(+) and CAA/tau(−) groups. In early‐phase PiB PET, patients with probable CAA who had tau(+) scans exhibited a non‐significantly lower SUVR in the whole cortex (*p* = 0.142; A) and significantly lower uptake in the temporal lobe (*p* = 0.043; B) than the tau(−) group. In the late‐phase scan, patients with CAA who had tau(+) scans had significantly higher SUVRs in the whole cortex (*p =* 0.049; C) and in the temporal lobes (*p* = 0.023; D) than the tau(−) group. The red dots indicate patients from the cognition cohort, and the blue dot represents patients from the ICH cohort.

Figure 2 shows representative dual‐phase PiB and late‐phase AV1451 PET images for the patients with CAA. In the early‐phase PiB scans, we observed a non‐significant difference in uptake for the whole cortex between the tau(+) and tau(−) groups (0.91 [0.88–0.96] vs. 0.95 [0.93–0.99], *p* = 0.142; Figure 3A). However, the tau(+) group had a significantly lower regional SUVR in the temporal lobe compared to the tau(−) group (0.87 [0.81–0.93] vs. 0.92 [0.89–0.98], *p* = 0.043; Figure 3B). There were no significant differences in the SUVRs for the frontal, parietal, occipital, precuneus, or PCC ROIs between groups in the early‐phase PiB scans (all *p* > 0.05; Table 2).

**TABLE 2:** Comparison of early‐ and late‐phase amyloid SUVRs between the tau(+) and tau(−) groups.

Parameter	Tau(+) (*n* = 20)	Tau(−) (*n* = 20)	*p*
Early‐phase PiB PET			
Whole cortex SUVR	0.91 (0.88–0.96)	0.95 (0.93–0.99)	0.142
Frontal lobe SUVR	0.89 (0.84–0.97)	0.93 (0.86–1.00)	0.265
Temporal SUVR	0.87 (0.81–0.93)	0.92 (0.89–0.98)	0.043
Parietal lobe SUVR	0.88 (0.81–1.00)	0.91 (0.88–1.01)	0.296
Occipital lobe SUVR	1.03 (0.95–1.13)	1.05 (0.99–1.14)	0.445
Precuneus SUVR	0.96 (0.87–1.01)	1.02 (0.95–1.66)	0.056
PCC SUVR	0.85 (0.71–0.89)	0.85 (0.73–0.94)	0.678
late‐phase PiB PET			
Whole cortex SUVR	1.65 (1.52–1.81)	1.47 (1.24–1.68)	0.049
Frontal lobe SUVR	1.65 (1.42–1.85)	1.51 (1.20–1.70)	0.081
Temporal lobe SUVR	1.57 (1.46–1.78)	1.42 (1.23–1.53)	0.023
Parietal lobe SUVR	1.80 (1.59–1.99)	1.59 (1.26–1.78)	0.043
Occipital lobe SUVR	1.69 (1.47–1.81)	1.45 (1.34–1.75)	0.134
Precuneus SUVR	1.93 (1.59–2.15)	1.69 (1.34–1.84)	0.072
PCC SUVR	1.60 (1.42–1.84)	1.68 (1.24–1.83)	0.989

*Note:* Values are median (interquartile range).

Abbreviations: PCC, posterior cingulate cortex; SUVR, standardized uptake value ratio.

Next, we compared the late‐phase PiB scans between the tau(+) and tau(−) groups. The tau(+) group had a significantly higher whole cortex SUVR than the tau(−) group (1.65 [1.52–1.81] vs. 1.47 [1.24–1.68], *p* = 0.049; Figure 3C) and higher SUVRs in the temporal lobe (1.57 [1.46–1.78] vs. 1.42 [1.23–1.53], *p* = 0.023; Figure 3D) and parietal lobe (1.80 [1.59–1.99] vs. 1.59 [1.26–1.78], *p* = 0.047). No significant differences were observed in the other ROIs (Table 2).

### 3.2 Associations Between Early‐ and Late‐Phase Amyloid Uptake and Tau Burden

To investigate whether amyloid PET parameters could predict tau PET uptake, we constructed linear regression models to examine the associations between these parameters and the meta‐temporal AV1451 SUVRs (Table 3). The early‐phase PiB SUVR of the temporal lobe demonstrated a significant negative correlation with the tau burden (*r* = −0.34, *p* = 0.032; Table 3, model 1). This correlation remained significant after adjusting for age (*r* = −0.34, *p* = 0.035, model 2) and after adjusting for age and cerebral amyloid load (i.e., late‐phase whole cortex PiB SUVR; *r* = −0.34, *p* = 0.038, model 3).

**TABLE 3:** Associations between amyloid scan parameters and meta‐temporal lobe tau burden.

Regions of interest	Model 1 (univariable)			Model 2 (age‐adjusted)			Model 3 (age‐ and d‐whole cortex SUVR‐adjusted)		
	*β* (95% CI)	*r*	*p*	*β* (95% CI)	*r*	*p*	*β* (95% CI)	*r*	*p*
eTemporal lobe	−1.17 (−2.24 to −0.10)	−0.34	0.032	−1.17 (−2.25 to −0.09)	−0.34	0.035	−1.10 (−2.14 to −0.06)	−0.34	0.038
dWhole cortex	0.29 (−0.02 to 0.60)	0.30	0.064	0.35 (0.02 to 0.67)	0.33	0.038	—	—	—
dTemporal lobe	0.32 (−0.02 to 0.67)	0.29	0.066	0.37 (0.01 to 0.43)	0.33	0.043	—	—	—
dParietal lobe	0.23 (−0.01 to 0.47)	0.30	0.064	0.27 (0.02 to 0.52)	0.33	0.039	—	—	—

Abbreviations: dWhole cortex, late‐phase whole cortex SUVR; eTemporal, early‐phase temporal lobe SUVR.

In addition, the late‐phase PiB SUVRs of the whole cortex, temporal lobe, and parietal lobe also showed strong trends or significant correlations with the tau burden in the univariable and multivariable models after adjusting for age (Table 3).

### 3.3 Diagnostic Value of Early‐ and Late‐Phase Amyloid PET for Predicting Tau Pathology

As the temporal lobe SUVRs of both the early‐ and late‐phase PiB scans significantly correlated with the tau burden, we investigated the diagnostic value of these parameters for identifying positive tau pathology in CAA (Table S1).

For the early‐phase SUVR of the temporal lobe, the cut‐off point of ≤ 0.872 determined by the Youden index had an AUC of 0.69 (95% CI: 0.52–0.82), sensitivity of 55% (95% CI: 31.5%–76.9%), specificity of 85% (95% CI: 62.1%–96.8%), PPV of 78.6% (95% CI: 54.6%–91.8%), and NPV of 65.4% (95% CI: 52.9%–76.0%). For the late‐phase SUVR of the temporal lobe, the optimal cut‐off of > 1.449 yielded an AUC of 0.71 (95% CI: 0.55–0.84), a sensitivity of 80% (95% CI: 56.3–94.3), specificity of 65% (95% CI: 40.8%–84.6%), PPV of 69.6% (95% CI: 54.7%–81.2%), and NPV of 76.5% (95% CI: 56.1%–89.2%). There were no significant differences between the ROC curves for the early‐phase and late‐phase SUVRs of the temporal lobe (*p* = 0.857).

**FIGURE 4:**
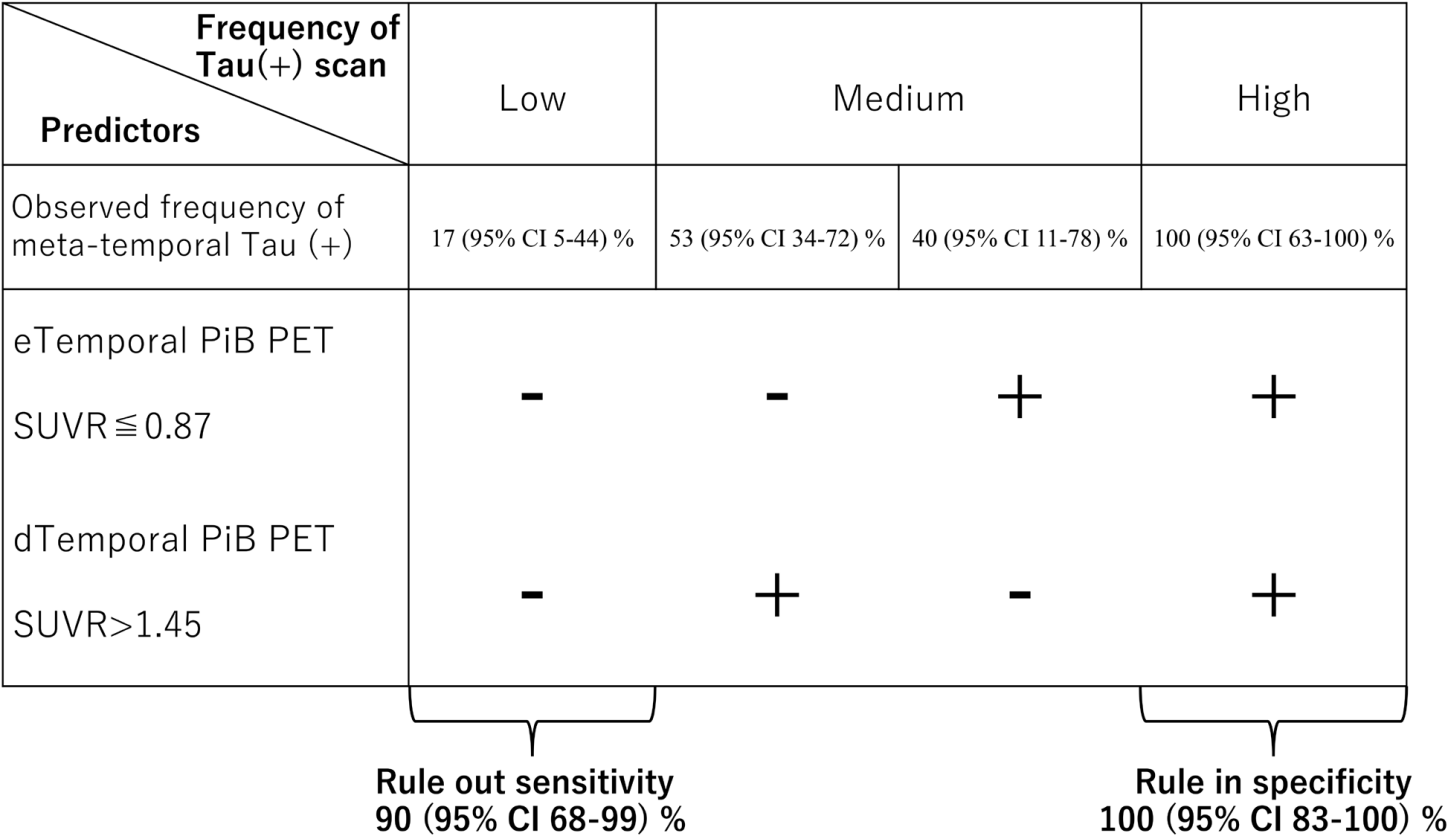
Combination of two PET markers for predicting tau PET(+) in CAA. We combined two PET biomarkers, reduced uptake in early‐phase temporal PiB PET (indicating reduced regional perfusion) and increased uptake in late‐phase temporal PiB PET (indicating increased amyloid load), to detect tau (+) in CAA. When both markers are absent, tau pathology in CAA can be ruled out with a sensitivity of 90% (95% CI: 68%–99%). When both markers are present, the specificity for tau positivity is 100% (95% CI: 83%–100%), which suggests that this combination of markers may help to effectively identify tau pathology in patients with CAA.

We next evaluated whether combining the cut‐off values for the early‐ and late‐phase SUVRs of the temporal lobe could provide reliable diagnostic value for tau pathology in CAA (Figure 4). Based on these cut‐offs, a negative early‐phase (above the cut‐off) and negative late‐phase amyloid scan (below the cut‐off) provided a sensitivity of 90% (95% CI: 68%–99%), suggesting that the absence of these markers in the temporal lobe could be used to confidently rule out CAA with tau pathology. Conversely, the combination of both a positive early‐phase (below cut‐off) and positive late‐phase scan (above cut‐off) provided a strong specificity of 100% (95% CI: 83%–100%), meaning that the presence of these markers in the temporal lobe could potentially be used to confirm the presence of CAA with tau pathology (Figure 4).

## 4 Discussion

In this prospective study, we investigated the findings of dual‐phase amyloid PET imaging in patients with CAA and compared the PET parameters between the tau(+) and tau(−) groups. We observed significantly lower regional uptake in the temporal lobe in the early‐phase scan and higher whole cortex and temporo‐parietal regional uptakes in the late‐phase scan in the tau(+) group. These findings suggest that CAA patients with tau markers have lower perfusion and an increased amyloid burden in the temporal lobe compared to CAA patients without tau markers. The early‐phase temporal lobe PiB SUVR independently correlated with the tau burden on PET. Overall, both early‐ and late‐phase amyloid PET scans had moderate‐to‐good diagnostic utility for predicting tau pathology in CAA, and our proposed combination of dual‐phase amyloid PET provided a reliable approach with a high rule‐out sensitivity (80%) and excellent rule‐in specificity (100%) in our series of patients.

Late‐phase or static amyloid PET indicates cerebral amyloid deposition status [10]. Early‐phase amyloid PET, that is, usually the images acquired in the first 6 min after tracer injection, has been considered a topographic or functional biomarker of cerebral perfusion [8, 9, 21]. A previous study showed that early‐phase PiB retention correlates with the relative cerebral blood flow (CBF) measured using ^15^O‐water PET, which suggests that early‐phase PiB PET could provide an accurate assessment of cerebral perfusion status [21]. Brain perfusion could also represent a proxy of cerebral metabolism due to neurovascular coupling; therefore, dual‐phase amyloid PET could be used to evaluate Aβ deposition and neurodegeneration with a single tracer injection [8]. To our knowledge, only one previous study conducted early‐phase amyloid PET in patients with CAA. Farid et al. compared the early‐phase PiB uptake in eleven patients with CAA without dementia [12]. The patients with CAA exhibited hypoperfusion in the occipital cortex compared to healthy controls or patients with AD, while perfusion in the posterior cingulate cortex (PCC) was relatively preserved in the patients with CAA compared to patients with AD. These findings are in line with another study that identified hypometabolism in the posterior cortex of patients with CAA compared to healthy controls using 18F‐FDG PET [22]. Our current study is the first evaluation of the relationship between early‐phase amyloid PET and a tau PET marker in CAA. We detected regional hypoperfusion in the temporal lobe in cases of CAA with meta‐temporal tau pathology. As increased tau‐related uptake in the temporal lobe on PET is considered to be an important AD signature [23, 24], our findings imply dual‐phase PiB imaging could potentially be used to assess concomitant AD in patients with CAA; this strategy may help to identify patients with CAA at high risk of neurodegeneration.

CAA is considered to be an age‐related cerebrovascular disease, with symptomatic lobar ICH being the most well‐known manifestation. However, cognitive impairment frequently occurs in CAA, and concomitant AD is present in some cases [5, 25]. Consistent with our own earlier reports that only included patients with CAA who presented with symptomatic ICH [7], we noticed higher total cSS scores and CAA scores in the tau(+) group than in the tau(−) group in this study. This finding suggests that tau pathology may be more frequently observed in severe CAA, especially in cases with leptomeningeal involvement. In addition to the concomitant AD, CAA itself may also be an independent factor that contributes to tau deposition. This hypothesis is supported by Kim et al. [26], who observed locally increased tau biomarker uptake around CMBs and cSS on PET. One potential explanation is that vascular amyloidosis may evoke an astrocytic response that results in tau aggregation [27, 28]. Whether the tau pathology in CAA represents a comorbidity of AD and how tau pathology interacts with vascular amyloid deposition are currently unknown. Therefore, the identification of tau pathology and the mechanisms by which tau affects the disease trajectory in CAA need to be investigated in future studies.

In this study, both early‐phase and late‐phase PiB uptake in the temporal lobe had good diagnostic ability for detecting tau pathology in CAA. Similarly to our findings for the early‐phase scan, Raman et al. showed that early‐phase hippocampal amyloid PET uptake exhibited a good correlation with and had predictive value for tau pathology in amyloid‐positive cognitively intact participants [29]. Studies performed in a memory clinic setting also reported that early‐phase amyloid PET could discriminate patients with AD from healthy controls, and the area of hypoperfusion in AD was most pronounced in the temporal lobe [8]. Our results further confirm there may be an association between early‐phase PiB PET changes in the temporal lobe and meta‐temporal tau signatures in patients with CAA. In our series, using the cut‐off for the temporal lobe on the early‐phase scan provided good specificity for detecting tau (85%), which suggests that early‐phase PiB PET scans could possibly provide a reliable way to rule out tau pathology in CAA cases with positive amyloid scans. Furthermore, our combined early‐ and late‐phase approach provided satisfactory rule‐out (90%) and rule‐in (100%) values for detection of tau pathology in CAA in our series. As sporadic CAA with and without tau pathology shows distinct clinical profiles and may require different management or monitoring strategies [6, 30], dual‐phase PiB scans may provide a clinically useful tool to identify high‐risk patients with CAA who may also have concomitant AD or be vulnerable to neurodegeneration.

Previous studies of PET in CAA were largely derived from late‐phase amyloid PET data. Compared to typical AD, at the group level, patients with CAA have lower whole cortex uptake; however, relative occipital uptake predominates [31, 32, 33, 34]. However, the lack of specificity of amyloid tracer binding to vascular Aβ compromises the clinical diagnostic utility of these markers to differentiate between CAA and AD, and the exact amyloid PET imaging features of CAA with concomitant AD remained largely unknown. The assessment of tau biomarkers has become another approach to delineate the potential concomitant AD pathology in CAA. While amyloid PET is becoming increasingly accessible worldwide [35], the availability of tau PET remains rather limited. This study highlights the potential utility of dual‐phase amyloid PET as an effective screening tool to identify CAA patients suspected of having concomitant tau deposition.

There are some limitations to this study. Firstly, we only compared the amyloid PET findings within a CAA cohort and did not include a “pure” AD group or age‐matched healthy controls. Thus, we cannot infer whether the dual‐phase amyloid PET pattern we identified in cases of CAA with concomitant tau is specific to CAA or is a consequence of combined CAA and AD pathology. Secondly, the diagnoses of CAA were based on the clinicoradiological Boston criteria but were not histologically confirmed. The Boston criteria provide good sensitivity and specificity for CAA in survivors of ICH; however, the diagnostic value of these criteria may be inferior in patients who have not suffered a hemorrhage [36]. Thirdly, we only included cases that were visually amyloid‐positive on PET in this analysis because, in clinical practice, AD‐signature tau might not be considered for patients with CAA who have a visually amyloid‐negative scan; these patients may represent a subgroup with a milder or regional form of CAA. Further research is required to determine whether, or how, to assess tau pathology that accompanies vascular Aβ but not parenchymal Aβ. Lastly, the sample size of the current study was relatively small, and larger‐scale studies are essential. However, to our knowledge, this is the largest prospective comprehensive dual‐phase amyloid PET dataset reported for a CAA cohort to date.

## 5 Conclusion

This study indicates dual‐phase amyloid PET scans may have significant diagnostic value for the differentiation of concomitant tau pathology in CAA. We demonstrate that meta‐temporal tau in CAA is associated with a higher amyloid load and reduced perfusion in the temporal lobe, which is suggestive of neurodegeneration. The combination of early‐ and late‐phase amyloid PET scans may provide a novel approach to detect tau pathology in CAA, and could also help reduce the potential radiation exposure and avoid additional scans or the expense of additional imaging for tau biomarkers.

## Author Contributions

Meng‐Ting Chiang and Hsin‐Hsi Tsai conceptualized the presented study. Meng‐Ting Chiang conducted the analysis of early‐phase PiB PET and drafted the initial manuscript. Chia‐Ju Liu and Ruoh‐Fang Yen analyzed the late‐phase PiB and Tau PET data and validated the analytical methods. Bo‐Ching Lee performed the MRI analysis for all patients. Hsin‐Hsi Tsai supervised the study's findings and revised the manuscript. All authors participated in result discussions and contributed to the final manuscript.

## Conflicts of Interest

The authors declare no conflicts of interest.

## Supporting information


Appendix S1.

## Data Availability

All data will be made available upon reasonable request to the corresponding authors.
