# Supplementary material for: Dual‐Phase C‐11 PiB PET Images for Detecting Tau Pathology in Cerebral Amyloid Angiopathy
Source: Ann Clin Transl Neurol. 2025 Mar 3;12(5):905–14. doi: 10.1002/acn3.70021 (PMC12093337; doi:10.1002/acn3.70021)
Supplement: Supplementary file 1 — Appendix S1. [file ACN3-12-905-s001.pdf]

Supplementary Figure 1. Histogram showing distribution of meta-temporal SUVR on AV1451 PET in probable CAA

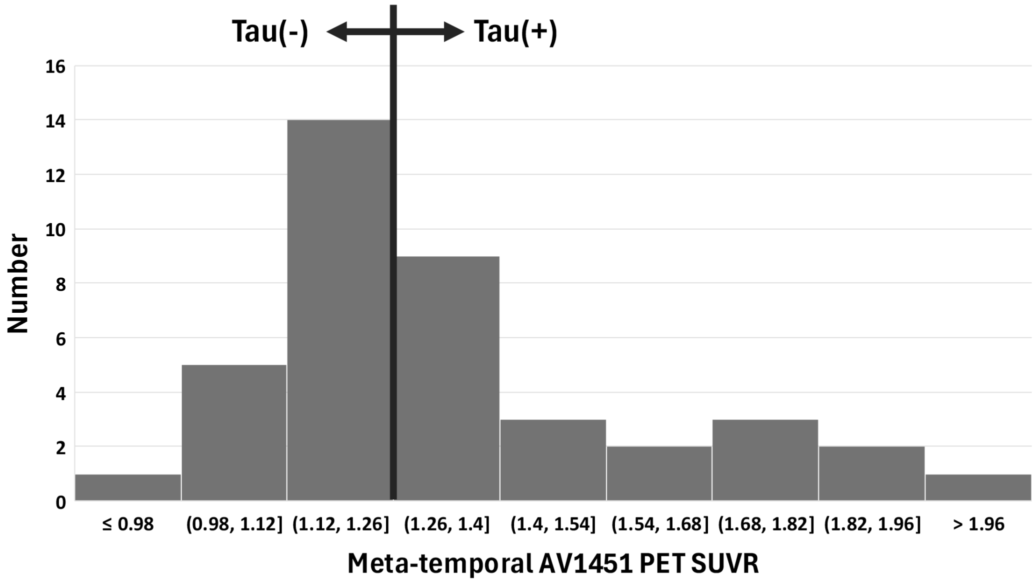

Supplementary Table 1. Diagnostic performance of early-phase PiB PET for predicting tau pathology in probable CAA

	eTemporal lobe SUVR	dTemporal lobe SUVR
**Cutoff value**	≤0.872	>1.449
**Sensitivity, %**	55(31.5-76.9)	80(56.3-94.3)
**Specificity, %**	85(62.1-96.8)	65(40.8-84.6)
**AUC**	0.69(0.52-0.82)	0.71(0.55-0.84)
**PPV, %**	78.6(54.6-91.8)	69.6(54.7-81.2)
**NPV, %**	65.4(52.9-76.0)	76.5(56.1-89.2)
**Significance level, *P***	0.030	0.015
**Diagnostic utility (accuracy), %**	70.0	72.5

eTemporal: early-phase temporal lobe SUVR; dTemporal: late-phase temporal lobe SUVR; AUC: area under the curve; NPV: negative predictive value; PPV: positive predictive value; SUVR: standardized uptake value ratio (relative to the pons).
